# Schwann cells in regeneration and cancer: an epithelial–mesenchymal transition perspective

**DOI:** 10.1098/rsob.240337

**Published:** 2025-03-05

**Authors:** Francisco Gracia, Berta Sanchez-Laorden, Jose A. Gomez-Sanchez

**Affiliations:** ^1^ Instituto de Neurociencias CSIC-UMH, San Juan de Alicante, 03550, Spain; ^2^ Instituto de Investigacion Sanitaria y Biomedica de Alicante (ISABIAL), Alicante 03010, Spain

**Keywords:** Schwann cells, epithelial–mesenchymal transition, plasticity, regeneration, cancer

## Abstract

In the peripheral nervous system, glial cells, known as Schwann cells (SCs), are responsible for supporting and maintaining nerves. One of the most important characteristics of SCs is their remarkable plasticity. In various injury contexts, SCs undergo a reprogramming process that generates specialized cells to promote tissue regeneration and repair. However, in pathological conditions, this same plasticity and regenerative potential can be hijacked. Different studies highlight the activation of the epithelial–mesenchymal transition (EMT) as a driver of SC phenotypic plasticity. Although SCs are not epithelial, their neural crest origin makes EMT activation crucial for their ability to adopt repair phenotypes, mirroring the plasticity observed during development. These adaptive processes are essential for regeneration. However, EMT activation in SCs-derived tumours enhances cancer progression and aggressiveness. Furthermore, in the tumour microenvironment (TME), SCs also acquire activated phenotypes that contribute to tumour migration and invasion by activating EMT in cancer cells. In this review, we will discuss how EMT impacts SC plasticity and function from development and tissue regeneration to pathological conditions, such as cancer.

**Figure:**
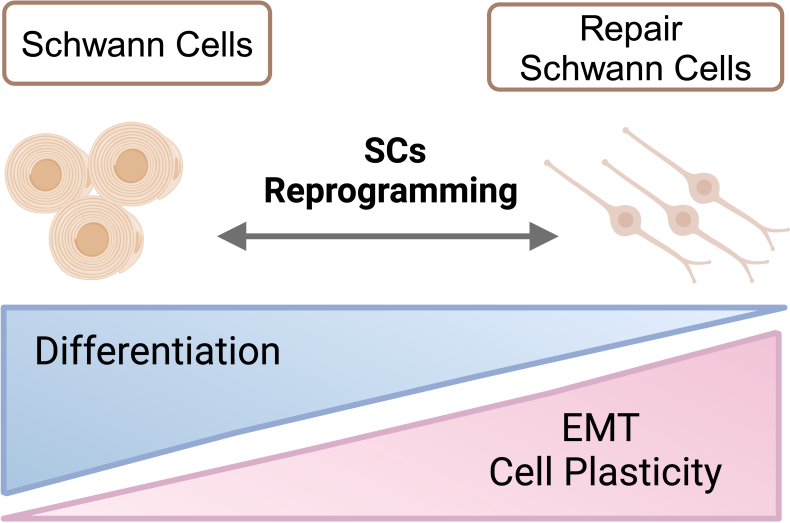

## 1. Introduction

In the peripheral nervous system (PNS), Schwann cells (SCs), glial cells derived from the neural crest, play a vital role in supporting and maintaining nerve function. A distinctive feature of SCs is their remarkable plasticity, allowing them to adapt dynamically to various physiological and pathological conditions [1]. This plasticity is essential for orchestrating regenerative processes such as digit tip regeneration, skin wound healing and peripheral nerve injury [2–4]. In these contexts, quiescent and adult SCs undergo cellular reprogramming, transforming into proliferating, progenitor-like cells that drive the regenerative response [5–7]. Specifically, during nerve damage these specialized cells, known as repair SCs, undergo a regulated phenotypic switch characterized by the downregulation of myelin-related genes and the upregulation of transcriptional mechanisms that support regeneration. Key players in this process include c-Jun, mitogen-activated protein kinase pathways, Sonic Hedgehog (Shh) and chromatin modifications that control and regulate the repair programme [7–12]. This adaptive reprogramming of the SCs is essential for creating a conducive environment for axonal regrowth and the restoration of nerve function.

However, the regenerative capacity of SCs can be corrupted in pathological conditions such as tumour innervation, neuropathies and pain [13–15]. In these situations, SCs undergo phenotypic changes with some similarities to those observed during nerve repair, enabling them to support these diverse processes and sustain damage. While the SC injury response, particularly in the context of nerve repair has been extensively investigated [6,16–18], the knowledge about systems regulating SC plasticity is still increasing. Interestingly, the activation of epithelial–mesenchymal transition (EMT) and the upregulation of stemness-associated genes have been observed in the response of SCs to injury [9,19]. However, the precise role of these programmes in SC plasticity remains under-investigated.

EMT is a complex biological process that enables epithelial cells to progressively lose their adherent phenotype and gain mesenchymal features. Classically, EMT has been associated with higher motility and invasiveness [20]. However, it has become clear that EMT is not a single process but includes different biological processes not only related to invasion. Furthermore, EMT is not always a binary switch between two distinct states; cells can also acquire an intermediate or hybrid phenotype that combines both epithelial and mesenchymal traits, known as partial EMT [21–23]. EMT is induced by signals from the microenvironment, such as TGFβ, which activate transcription factors (TFs) and miRNAs that repress epithelial identity and induce the mesenchymal programme [24,25]. These TFs, known as EMT-TFs, include members of the Snail, Zeb and Twist families [21]. EMT is crucial in several developmental processes, including gastrulation, neural crest formation and organogenesis [26], and in adulthood, it plays a critical role in physiological conditions such as wound healing. During tissue injury, reactivation of EMT promotes repair in adult vertebrates by inducing dedifferentiation, cell plasticity and enabling cell cycle re-entry. In this way, cells acquire a transient progenitor state that allows them to migrate to injury sites, contribute to wound healing and restore tissue integrity [23]. However, the expression of EMT-related genes can be reactivated in pathological conditions such as fibrosis and cancer [27,28]. In cancer, for instance, EMT can be hijacked by tumour cells to delaminate from the primary tumour and migrate, a process essential for metastasis [20,29].

Although SCs are not epithelial cells, they derive from neural crest precursors and increasing evidence suggests that the activation of EMT contributes to their ability to dedifferentiate and adopt repair phenotypes, mimicking the plasticity seen during development. The activation of this programme in SCs is biologically significant because EMT-induced changes typically provide cells with increased motility and morphological flexibility, essential properties during extensive tissue remodelling that occurs at injury sites [9,19]. Furthermore, the expression of EMT-related genes is not only important for SCs differentiation and physiological role but has also been associated with situations that include infections and cancer.

This review gives an overview of the current state of the art on the contribution of EMT to SC plasticity and function. From SC development and contribution to nerve repair and tissue regeneration to the knowledge of pathological conditions such as cancer.

## 2. Dynamic epithelial–mesenchymal transition processes in neural crest development

During the development of a multicellular organism, morphogenetic movements enable cells to acquire migratory and invasive properties facilitating the correct formation of tissues and organs at the proper location [26]. This process involves dynamic transitions between epithelial and mesenchymal phenotypes through the activation of the EMT programme [20]. The ability of cells to switch between these states has been crucial for evolution enabling the generation of cells with highly versatile behaviours [30]. Except for the central nervous system (CNS) and the epidermis, all organs and tissues result from one or several rounds of EMT during ontogeny. The initial rounds, known as primary EMTs, include the formation of parietal endoderm and the delamination of cells from the primitive streak at gastrulation and of the neural crest cells (NCCs) from the neural tube [20].

In particular, the neural crest is an embryonic stem cell population specific to vertebrates that is located in the dorsal part of the neural tube. NCCs undergo a tightly regulated EMT through modification of molecular pathways that enhance their migratory capabilities. While some NCCs remain in the neural tube, forming a stem cell niche [31], many others delaminate, leaving the neuroepithelium to migrate away towards their final destination. EMT plays a pivotal role in this process since the loss of epithelial properties allows the pre-migratory NCCs to detach from adjacent neural tube epithelial cells. In addition, the acquisition of mesenchymal features facilitates their extensive migration throughout the developing embryo [32,33].

During neurulation, NCCs activate sequential gene regulatory networks that include the induction of EMT-TFs such as Snail1/2, Twist and Zeb [34–36]. As a consequence of EMT activation, NCCs modulate the composition of the plasma membrane, which is crucial for persistent migration. One important change in the NCC membrane is the cadherin switch from type 1 cadherins (including E-cadherin), which are crucial for epithelial adhesion, to more mesenchymal type II cadherins (including cadherin-7 and cadherin-11) [37]. The regulation of cadherin expression by TFs is crucial for EMT. Snail1/2 are able to directly downregulate the expression of type I cadherins by binding to the E-cadherin promoter [38]. Furthermore, Snail1/2, along with Lmo4, are implicated in the downregulation of N-cadherin and the type II cadherin-6B, the expression of which decreases during delamination [39]. The TF Sox9 also cooperates with Snail2 to drive EMT [40]. During development, Wnt/β-catenin signalling plays multiple roles in regulating neural crest EMT by modifying Snail expression, which in turn suppresses E-cadherin expression [41].

Nevertheless, it is important to note that not only Snail TFs are important for this process but also Zeb2 and Twist are essential to downregulate E-cadherin and allow the cadherin switch to occur [42]. All these processes confer NCCs migratory capabilities that differ along the body axis. Indeed, NCCs acquire different degrees of mesenchymalization resulting in different modes of migration [43]. While trunk NCCs delaminate as individuals, cranial NCCs migrate in a more collective manner reminiscent of cells undergoing partial EMT [44]. Interestingly, signals that trigger EMT in the neural crest include interactions with adjacent tissues and the extracellular matrix (ECM). For instance, NCCs secrete proteases such as MMP-2, MMP-9 and MMP-14, which are necessary for cell migration, in order to break cadherin junctions and exit the neuroepithelium [37,45–47].

The activation of EMT in neural crest development is crucial not only for driving its significant migratory behaviour but also for influencing its pluripotency [23]. NCCs can differentiate into several cell types. In the cranial region, NCCs give rise to early ectomesenchyme, which subsequently differentiates into adipocytes, chondrocytes, osteocytes, smooth muscle cells and dermal fibroblasts of the face, among others. In the trunk, the neural crest progeny includes melanocytes, sensory and autonomic neurons and glial cells, including SCs [48–51]. Moreover, EMT can promote NCC fate changes. This process is important since it decreases the ectodermal/neuronal identity that enables the acquisition of the ability to produce mesenchymal derivatives. Consistent with this, the deletion of the EMT-TF Twist1 in cranial neural crest progenitors reduces their ability to produce these mesenchymal derivatives without any impact on the production of ectodermal ones including neural and glial cells [52,53].

Collectively, the dynamic and complex process of EMT is essential during NCC delamination, migration and differentiation. Therefore, perturbation of neural crest EMT genes, including the EMT-TFs Snail, Twist and Zeb2, can affect the delamination and migration of these cells resulting in several birth defects, referred to as neurocristopathies [54]. Furthermore, the similarities between developmental EMT and the pathological EMT, reactivated in conditions such as fibrosis and cancer, suggest that an in-depth understanding of neural crest EMT could lead to the development of novel therapies and treatments for these diseases.

## 3. Schwann cell plasticity in physiology

### 3.1. Schwann cell differentiation

As the embryo develops, some migratory trunk and cranial NCCs differentiate into Schwann cell precursors (SCPs), representing the initial stage of SC lineage. SCPs are intimately associated with the developing nerves of the PNS, migrating along and proliferating on their surfaces [55,56]. These glial precursors distribute throughout the body and give rise to a variety of cell types including melanocytes [57], endoneural fibroblasts, parasympathetic and enteric neurons [58,59], mesenchymal stem cells [60] and different subtypes of mature SCs [61].

In light of the extensive range of SCP-derived fates, SCPs could be considered as nerve-associated states of NCCs that continue into later developmental stages. This hypothesis is supported by the findings of Kastriti *et al*. [62], who reported that both early SCPs and late NCCs share a multipotent ‘hub’ state that contains differentially biased cells [62]. A distinctive feature of SCPs is their reliance on axonal signals for survival, as evidenced by the observation that axonal degeneration induces SCP death in both *in vivo* and *in vitro* settings. In particular, neuregulin 1 from axons has been shown to be essential for SCP survival and proliferation [63,64]. Furthermore, SCPs can be distinguished from NCCs by specific changes in gene expression that are tightly regulated. While some of these genes, whose expression is upregulated in SCP, are unique to this stage (e.g. cadherin 19) [65], others such as Sox10 persist into later developmental stages of the SC lineage [66,67].

Mature SCs do not arise directly from SCPs; rather, they emerge from an intermediate state. Once SCPs are driven towards the SC lineage, SCP develops into immature SC (iSC) that, depending on axonal signalling, differentiate into non-myelinating SC (or Remak SCs) or promyelinating SC that finally transform into myelin-forming SC [68]. Unlike SCPs, the survival of iSCs is no longer axon-dependent [69], and their molecular signature evolves, with increased expression of genes such as Gfap and S100β. Therefore, the development of SCs is orchestrated by a complex and coordinated set of genes and non-cell-autonomous inputs that guide proper differentiation, migration and proliferation [70].

**Figure 1.**
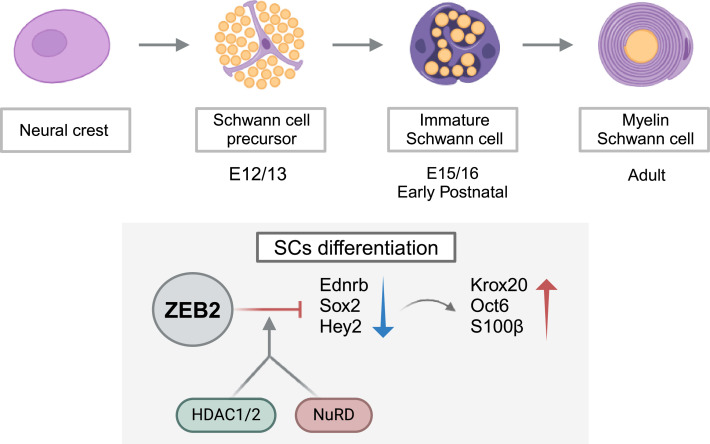
Role of the EMT-TF Zeb2 in SC development. In SCs, Zeb2 is transiently expressed during key embryonic stages, from NCCs to iSCs. During the progression of the SCs lineage, Zeb2 recruits histone deacetylases 1 and 2 and NuRD chromatin-remodelling (HDAC1/2-NuRD) complexes to act as a transcriptional repressor of differentiation inhibitors such as Ednrb, Sox2 and Hey2. Consequently, genes encoding for myelin proteins like Oct6, Krox20 and S100β are upregulated, which is essential for appropriate SC differentiation and myelination (figure created with BioRender.com).

Given that SCs originate from NCCs, it is not surprising that factors associated with EMT could play a critical role in regulating SC lineage development. One of the factors that has been reported to be particularly important during nervous system development is the EMT-TF Zeb2. Indeed, Zeb2 knock-out mice embryos die due to the failure of neural tube closure and defects in the neural plate [71,72]. In humans, Zeb2 aberrant expression caused by mutations triggers important neurodevelopmental disorders including Mowat–Wilson syndrome, which is characterized by mental retardation, craniofacial abnormalities and Hirschsprung’s disease [73]. In SCs, Zeb2 is transiently expressed during development, and it is reactivated in mature SCs after nerve injury [74]. Several studies have demonstrated that Zeb2 acts as a transcriptional repressor in SCs regulating a number of signalling pathways previously identified as essential for SC development [74,75]. One of these is endothelin signalling, which plays a crucial role in the development of neural crest derivatives. Following transcriptome analysis of Zeb2-deficient SCs in peripheral nerves, the endothelin receptor B (Ednrb) was identified as one of the target genes that Zeb2 can repress. Also during SC development, Ednrb activation has been observed to delay the formation of iSCs from SCPs both *in vitro* and *in vivo* [76]. Another important factor regulated by Zeb2 is Notch signalling, which is essential during several stages of SC development [77]. When Zeb2 is absent, Notch signalling is constitutively activated in sciatic nerves, blocking SC differentiation and therefore myelination. In particular, Zeb2 negatively regulates Hey2, which is a downstream effector of Notch signalling [78], inducing the expression of Krox20 (also called Egr2) a TF essential for SC myelination [74] (figure 1).

Sox2 is another negative regulator of SC maturation that seems to be regulated by Zeb2 [74]. Sox2 is known as an inhibitor of myelination that is downregulated in early SC development at the same time that Krox20 is expressed [79]. In fact, enforced expression of Sox2 in SCs leads to persistent proliferation of SCs and inhibition of SC maturation. Furthermore, Zeb2-deficient SCs significantly upregulate Sox2 levels maintaining SCs in a progenitor-like state by suppressing differentiation markers including Krox20 [79]. Given that Sox2 is downstream of Notch signalling in some contexts [80], it is possible that Notch-Hey2 activation may promote and maintain Sox2 expression to inhibit differentiation in Zeb2-deficient SCs. Wu *et al*. [75] also reported that Zeb2 recruits histone deacetylases 1 and 2 and NuRD chromatin-remodelling (HDAC1/2-NuRD) complexes, which then suppress the myelination inhibitors Hey2 and Sox2 during SC differentiation (figure 1).

These findings indicate that at early stages of SCs development, the EMT-TF Zeb2 has a crucial role in facilitating differentiation by repressing genes such as Ednrb, Sox2 and Hey, which must be downregulated to facilitate axon sorting and myelination.

At later stages, post-transcriptional modifications are also essential for the appropriate development of the SC lineage. In particular, an adenosine-to-inosine RNA editing process, which is catalysed by ADAR protein family members including ADAR1, plays an essential role in the transition from promyelinating SC to myelin-forming SCs [81]. Adar1 is the most highly expressed ADAR outside the CNS [82] and is critical for editing endogenous dsRNAs as self to prevent their immune recognition [83]. In SCs, Adar1 deletion has a severe impact on SCs differentiation, resulting in the absence of myelin formation. RNA-seq analyses of Adar1-deficient sciatic nerves showed that SCs are blocked at the promyelinating stage being unable to myelinate [81]. Consequently, the maintenance of a more progenitor-like state in Adar1-deficient SCs caused the increased expression of EMT-related genes critical for neural crest development. Upon Adar1 deletion, an increase in the expression of the master regulators Twist1 and 2, Snail2, Lef1 and Met was observed, accompanied by Cdh1 downregulation. However, other classical mesenchymal genes such as Cdh2 and Vimentin were not altered, suggesting that only part of the EMT programme is activated after Adar1 deletion [81].

All these studies indicate that the EMT programme as well as other important pathways for the development of SC lineage must be tightly regulated for correct SC differentiation and myelination. However, further studies are needed to understand the role of different genes involved in SC plasticity including other EMT-TFs during development. These studies would provide us with a better understanding of the outcome of demyelinating neuropathies and other diseases of the PNS.

### 3.2. Tissue regeneration

In adult organisms, the proliferative abilities of most tissues and organs are notably restricted, necessitating the development of mechanisms to repair and restore functions upon injury [84]. One such mechanism is the EMT, which has been identified as a standard physiological response after injury that contributes to the tissue repair process (extensively reviewed in Youssef & Nieto [23]). This is not unexpected, given that dedifferentiation is a key mechanism associated with natural regenerative capabilities. Dedifferentiation is defined as the regression of differentiated somatic cells to a less specialized state that is more compatible with cell cycle re-entry. This reversion allows the generation of a pool of reparative cells, which subsequently undergo re-differentiation to replace cells lost during injury [85].

The phenomenon of injury-induced EMT has been observed in a multitude of contexts, including zebrafish heart, skin wounds and kidney tubules [21,23,86]. For instance, in cutaneous wounds, epithelial keratinocytes initiate a partial EMT characterized by the upregulation of the Snail2 TF, enabling the collective migration of cells towards the wound boundary [87,88]. Similarly, in the kidney, both obstruction and chemical injury induce partial EMT in tubular epithelial cells, a process that relies on the activation of Snail1 [28,89]. These cellular responses resemble the reprogramming events observed in the generation of SCs post-injury, illustrating a conserved cellular response to damage.

SCs are crucial not only for peripheral nerve repair but also contribute to regenerative processes [14,90]. Although research in this area remains limited to a few tissues, there is evidence suggesting that adult SCs may retain some degree of the multipotency observed in SC precursors that can contribute to the regeneration process [91–93]. This plasticity of SCs is essential for maintaining stemness and creating a supportive microenvironment for stem cell expansion, which promotes tissue regeneration [94].

A clear example of the importance of SCs in regeneration is observed in the regrowth of appendages in amphibians. Salamanders possess extraordinary regenerative properties, with the capacity to regenerate entire limbs following amputation. This process begins with the formation of a blastema, a mass of proliferating stem-like cells that reconstruct the lost limb [95]. While it has long been known that successful limb regeneration is primarily nerve-dependent, more recent studies have clarified the crucial role of repair SCs in the efficacy of the regeneration process. These cells provide essential trophic support to sustain blastema proliferation by secreting the PROD1 ligand and the newt anterior gradient (nAG) protein, which are critical factors for maintaining blastema cell proliferation through the PROD1 receptor. In fact, if the limb is denervated before amputation, nAG secretion by SCs is disrupted, leading to failed regeneration due to inadequate blastema expansion [96,97].

While these studies highlighted the potential role of SCs in regeneration outside the nervous system, more recent research has also exemplified their diverse involvement in tissue repair in mammals. Similar to limb regeneration in amphibians, the digit tip in mammals can regenerate through mechanisms that are also partially dependent on innervation [98,99] and SCs with a repair phenotype [2]. A mouse model of digit tip regeneration showed that mesenchymal repair progenitor cells, which populate the digit blastema, are able to proliferate and differentiate thanks to the essential paracrine factors oncostatin M and platelet-derived growth factor AA secreted by repair SCs [2]. Similarly, SCs also play a crucial role in bone repair. Using a mouse model of mandibular denervation, the same paracrine factors secreted by SCs during digit tip repair are also required for the skeletal stem cells to regenerate the mandibular bone. The denervation of the bone led to a reduction in SC numbers, decreasing the secretion of SCs paracrine factors and thereby impairing bone repair. This phenomenon is partially rescued when SCs with an activated phenotype are transplanted into the bone or when SC-derived growth factors are supplied [100].

Injury-activated SCs also play an important role in promoting wound healing in the skin [3,101]. The ability of SCs to dedifferentiate is required for their migration into the wound bed, enabling the proper regeneration of the dermis. In fact, specific genetic ablation of injury-activated SCs delayed wound contraction and closure by decreasing both epithelial proliferation and myofibroblast contraction, which are required for wound repair [102]. As in other repair contexts, SCs modulate the phenotype and behaviour of other cell types involved in the regenerative process through the secretion of paracrine factors that promote TGFβ to enhance the myofibroblast phenotype within the wound area [3]. Specifically, TGFB3 is the key TGFβ ligand that injury-activated SCs express in an early stage of wound healing, which is essential for the migration of keratinocytes and fibroblasts [103]. The activation and dedifferentiation of SCs following a skin injury have been found to occur at an early stage of the healing process by inducing the expression of the Wnt pathway. Intriguingly, SCs also modify their secretomes at different stages, suggesting that their plasticity is required for orchestrating diverse functions during wound regeneration [103].

Considering this, the remarkable plasticity that enables SCs to undergo a certain degree of dedifferentiation and acquire a repair phenotype following injury is considered to be the key factor promoting repair [7]. Although the underlying mechanism by which SCs become repair cells after nerve injury is better understood, whether SCs display distinct repair processes in different tissues remains unclear.

### 3.3. Peripheral nerve repair

Upon peripheral nerve injury, the loss of axonal contact results in the reprogramming of myelinating and Remak SCs into repair SCs, which allows PNS regeneration. These injury-activated SCs perform specific functions that are not carried out by fully differentiated SCs or developmental SCs. This includes the clearance of myelin and axonal debris, activation of innate immune responses and formation of Büngner bands, which allows axonal regrowth towards peripheral targets. This transition of differentiated but plastic SCs into repair SCs needs a reprogramming of the transcriptome leading to the suppression of genes related to myelination and activation of an injury-specific programme of gene expression [6,7,14,16]. As mentioned above, even though repair SCs are not considered to be epithelial cells recent studies show that upon nerve injury, these cells activate factors controlling the EMT programme and upregulate genes associated with stemness, including those crucial for self-renewal. This coordinated regulation of gene expression pathways promotes tissue remodelling and repair in a manner analogous to that observed in epithelial contexts [9,16,19,104].

**Figure 2.**
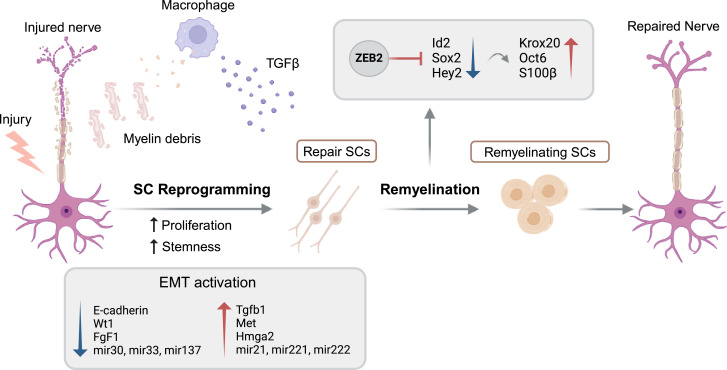
Involvement of EMT in SCs reprogramming upon nerve injury. In peripheral nerves, adult SCs respond to injury by activating repair-specific gene programmes that allow PNS regeneration. During this reprogramming, repair SCs activate EMT-related factors and upregulate genes associated with stemness, including those crucial for self-renewal. The activation of the EMT in repair SCs is induced by localized TGFβ whose expression is mainly increased by infiltrating macrophages upon nerve injury. During the remyelination phase of nerve regeneration, the EMT-TF Zeb2, which is reactivated in repair SCs after nerve damaged, plays an essential role. Zeb2 is responsible for inhibiting the expression of Ednrb, Id2 and Hey2, leading to the upregulation of myelin-related genes such as Krox20, Oct6 and S100β, which enable successful SC re-differentiation and remyelination. This repression reflects the analogous role that Zeb2 plays during the myelination phase in nerve development (figure created with BioRender.com).

EMT is a process that can be regulated by multiple factors including secreted signals, such as TGFβ family members, miRNAs including the miR-200 family and EMT-TFs that specifically bind to DNA in order to induce the expression of mesenchymal genes and repress epithelial ones [21]. In particular, the expression of TGFβ that is a very well-known inducer of the EMT [24] is increased after nerve injury in SCs, endoneurial fibroblasts and infiltrating macrophages [105]. In an *in vitro* model using RSC96 SCs, treatment with TGFβ1 enhanced their migratory and invasive capabilities through the activation of metalloproteinases MMP-2 and MMP-9 [106]. Similarly, *in vivo* experiments indicate that TGFβ1-treated SCs exhibit improved axonal regeneration capabilities [105]. The targeted deletion of TGFβ receptor II (TGFBR2) in SCs has clarified the specific role of TGFβ in promoting SC migration from the proximal nerve stump across the bridging region, to re-establish connections with the distal stump [19]. As a consequence of the localized TGFβ secretion in the injury site, SCs within the bridging region exhibit a clear EMT signature, which is characterized by an enhanced proliferation rate and a mesenchymal phenotype [19] (figure 2), reflecting the well-established link between EMT and cellular stemness [107,108]. However, despite TGFBR2 depletion in SCs, the long-term regeneration of nerves is normal, suggesting redundancy or additional compensatory mechanisms involved [19]. Clements *et al*. also showed the upregulation of the Myc stemness and Core pluripotency modules and the downregulation of polycomb-related factors in these cells. Furthermore, SCs in the distal stump have a less evident but still detectable EMT response. In support of this, an additional study performing RNA-sequencing analysis has identified increased levels of mRNAs and miRNAs associated with the EMT phenotype in mouse distal nerve samples [9]. This expression profile includes decreased expression of epithelial genes, such as E-cadherin, Wt1, Fgf1, Ndrg1, mir30, mir33 and mir137, along with an increase of EMT-related factors including Tgfβ1, Met, Hmga2, mir21, mir221 and mir222 (figure 2). Further findings from this study show an enrichment of EMT signatures among c-Jun transcriptional targets, key activator of the SC repair programme [9]. A subset of these genes, including Notch1, Shh and members from the miR-34, miR-221 and miR-222 families have previously been shown to contribute to repair SC function [77,109–111]. All these findings suggest that external factors can directly modulate intrinsic cellular factors such as EMT programmes in SCs to adapt local cell function to the specific requirements of the neighbouring tissue to ensure successful regeneration.

Analogously to the situation during SC development, another important EMT-related factor that plays a key role in nerve regeneration is Zeb2. During EMT, Zeb2 inhibits the expression of several genes linked to cell adhesion molecules, including E-cadherin [112]. Although Zeb2 is not expressed in mature SCs, an increased expression of Zeb2 is observed in repair SCs upon peripheral nerve injury. Initial evidence suggests that Zeb2 is not responsible for the immediate post-injury regulation of c-Jun and Sox2, meaning that its role is not critical for the initial formation of repair SCs. Nevertheless, Zeb2’s function becomes essential during the remyelination phase of nerve regeneration. At this later stage, Zeb2 downregulates the expression of negative regulatory elements such as Sox2 and Id2, as well as the Notch pathway effector Hey2 (figure 2). This repression is critical for the proper myelination of regenerating nerves, reflecting the analogous role that Zeb2 plays during the myelination phase in nerve development [74,75].

In a recent study, Daboussi *et al*. [113] show that the melanocyte-inducing transcription factor (MITF) is also essential for two critical aspects of SC plasticity during nerve injury: the transition of mature SCs into repair cells that maintain axonal integrity, and the subsequent re-differentiation of these repair cells into myelinating SCs that restore normal nerve conduction. This is attributed in part to the role of MITF in regulating the transcriptional repair programme in SCs, which influences SC dedifferentiation, metabolic and trophic factors as well as EMT. Following sciatic nerve transection, RNA sequencing in MITF mutant SCs revealed a deregulation of genes involved in cell adhesion and ECM composition both processes related to EMT, as well as signalling molecules including TGFβ and WNT signalling components. As a consequence, MITF mutant SCs exhibit reduced migratory capacity and increased adhesion properties, which are detrimental to EMT processes and cellular migration essential properties also in the context of wound healing [113].

## 4. Exploiting Schwann cell plasticity in disease

### 4.1. Intracellular pathogens

Intracellular pathogens, such as *Mycobacterium leprae* (ML), which is responsible for human leprosy, can exploit the remarkable plasticity of SCs to aid their dissemination [114–116]. This pathogen establishes infection in SCs, a primary non-immune target, resulting in neurological injury and sensorimotor loss [117–119]. Research by Masaki *et al*. [104] demonstrates that leprosy bacteria induce adult SCs reprogramming into progenitor/stem-like cells (pSLC) with mesenchymal and migratory properties, promoting bacterial spread to distal tissues. Infected SCs downregulate genes associated with differentiation, myelination and lineage while upregulating numerous developmental genes mainly related to mesoderm development, including homeodomain/Hox, EMT and neural crest-related genes. Key EMT regulators Twist1 and 2, Snail2 and Msx2, which induce EMT in epithelial cells [107,120], are among the highly upregulated TFs in ML-infected SCs. They also show that this switch in the SC fate is epigenetically regulated. In pSLC, most CpG sequences in the promoter regions of mesodermal/EMT genes such as Twist1, Prrx1, Tbx18 and Bmp6 exhibit significantly lower methylation compared to SCs. This suggests that the epigenetic status of these genes has shifted from a repressed to an active state in pSLC confirming epigenetic changes in key regulatory genes involved in the reprogramming of infected SCs [104]. This is an excellent example to highlight how the plasticity of adult SCs can be exploited not only for tissue repair processes but also in a pathological context such as infections by pathogens.

### 4.2. Epithelial–mesenchymal transition activation in tumours

In pathological conditions such as cancer, the reactivation of developmental programmes usually silent in adulthood is common. As such, EMT reactivation gives tumour cells the ability to detach from the primary tumour and invade the surrounding tissue. This allows them to invade blood or lymphatic vessels and metastasize to other organs [21]. As in physiological EMTs, pathological EMT also shows a great complexity depending on the tissue context. The aberrant expression of different EMT-TFs has been frequently observed in the development of various cancer types, particularly carcinomas and is often associated with poor prognosis and high risk of metastasis [20,29]. The plasticity of tumour cells in aggressive carcinomas is in part given by the dynamic and reversible nature of EMT pathways that facilitate the oscillation between proliferative and invasive cellular states [121,122]. Importantly, EMT also results in the acquisition of stem cell-like properties including slow proliferation and self-renewal potential contributing to tumorigenesis and therapy resistance [107,123].

EMT-like processes have been observed not only in carcinomas but also in non-epithelial cancers, such as glioblastoma, lymphoma, myeloma, leukaemia, sarcoma and melanoma [124]. In particular, melanoma cells, which derive from melanocytes and share the same developmental origin as SCs, reactivate embryonic programmes during transformation and can reversibly switch between different phenotypic states to favour progression [125]. As in SCs, this remarkable degree of plasticity is associated with the activation of EMT programmes and the mesenchymal phenotype is linked to metastasis and resistance to therapies [126–128].

Transcriptome analyses have revealed that mesenchymal melanoma cells resemble epithelial tumour cells after undergoing EMT. They almost lose their melanocytic properties [129,130] and express high levels of TGFβ-regulated genes and reduced E-cadherin levels [131,132]. Furthermore, EMT-TFs such as Zeb1 and Twist1, which are known to promote invasion and ECM changes, are present in mesenchymal-like melanoma cells [126] and play roles similar to those in EMT: they suppress E-cadherin, drive dedifferentiation and correlate with lower metastasis-free and recurrence-free survival rates [128,133]. However, there are also notable differences between melanoma phenotype switching towards a mesenchymal phenotype and EMT. For example, Snail2, which typically supports a mesenchymal phenotype in epithelial tumours, acts as a marker for melanocytic identity during both the development of the neural crest and the progression of melanoma [134]. Similarly, Zeb2, which is recognized as a mesenchymal marker in the context of epithelial cancers, does not promote invasion in melanoma but rather induces melanocytic differentiation [135]. Consequently, the phenotypic switch observed in melanoma is, at least in part, the result of a reprogramming process involving the expression of different EMT-TFs. This process is characterized by a progressive loss of Zeb2/Snail2 and a gain in Twist1/Zeb1 expression, which occurs as the melanocytes transition to malignant melanoma [128,136]

In line with the situation in melanoma and compatible with their common origin, transformed SCs also activate EMT programmes. SCs and their precursors can transform into benign, slow-growing nerve sheath tumours, denominated plexiform neurofibromas (PNs), which typically occur in individuals with neurofibromatosis type 1 (NF1) [137]. SCs are considered the primary transformed cells in PNs due to their biallelic mutation of the NF gene and their inherent invasive and angiogenic properties [138,139]. The NF1 gene encodes neurofibromin, a GTPase-activating protein (GAP) that enhances the intrinsic GAP activity of RAS proteins. Consequently, mutation or loss of NF1 leads to the activation of signalling through the RAS pathway [140]. In transformed epithelial cells, Ras signalling has a crucial role in cell proliferation and the induction and maintenance of EMT. In this context, constitutive activation of Ras, resulting from NF1 suppression, collaborates with the TGFβ pathway to induce EMT-related factors [25,141]. Compatible with this, in SCs, the loss of neurofibromin leads to increased expression of EMT-TFs such as Snail1, Slug, Twist, Zeb1 and Zeb2, both in cultured cells and in NF1-associated neurofibroma samples. The upregulation of these EMT inducers in NF1-deficient SCs promotes cell migration and invasion [142]. These observations indicate that neurofibromin is essential for the repression of EMT-related TFs and that the EMT signalling pathway plays a role in the development of neurofibromas following NF1 gene inactivation [142].

Furthermore, the hyperactivation of the Ras signalling pathways in NF1-deficient SCs results in an enhanced production of reactive oxygen species (ROS) [143]. While the impact of ROS on EMT appears to be dependent on cell types, particularly in SCs, it participates in the induction of the EMT programme [144]. Indeed, the use of lipoamide, which is a potent antioxidant [145], significantly impaired the invasive capacities of SCs, independently of NF1 expression. That suggests not only that NF1 loss induces EMT in transformed SCs but also that spontaneous EMT can occur upon ROS production [144]. However, further studies are required to elucidate the specific molecular mechanisms.

Furthermore, recent research has also revealed that the modulation of EMT processes in PNs involves the protein tyrosine phosphatase receptor S (PTPRS) [146]. Initially identified for its critical role in neural development [147], PTPRS has now been recognized for its broader impact on neurogenic tumours. Particularly in PN tissue, this receptor is frequently downregulated, a condition that has been associated with tumour recurrence. *In vitro* experiments have shown that a decrease in PTPRS expression contributes to enhanced migration and invasion of NF1-deficient SCs, as well as reduced expression of E-cadherin and increased N-cadherin levels. These findings suggest that PTPRS could influence the progression of PNs by regulating the EMT process [146].

Given the high degree of plasticity of SCs, it is not surprising that PNs exhibit cellular heterogeneity [148]. Indeed, multiple populations of SCs and their progenitors are crucial for neurofibroma formation. In a genetically engineered mouse model, the loss of function of Nf1 in nerve glial cells at the SCP stage is sufficient to promote PNs [137,149]. Taking advantage of the progression of single-cell RNA-sequencing technology, Kershner *et al*. [150] were able to define the PN transcriptome at single-cell resolution in a mouse model and human clinical samples. The researchers identified extensive changes in multiple SC populations that occur over time in PNs. In particular, non-myelinating SCs and SCP-like cells were notable for their continued EMT response in tumours, although this induction was not as evident as the EMT response in SCs in case of nerve injury [150].

**Figure 3.**
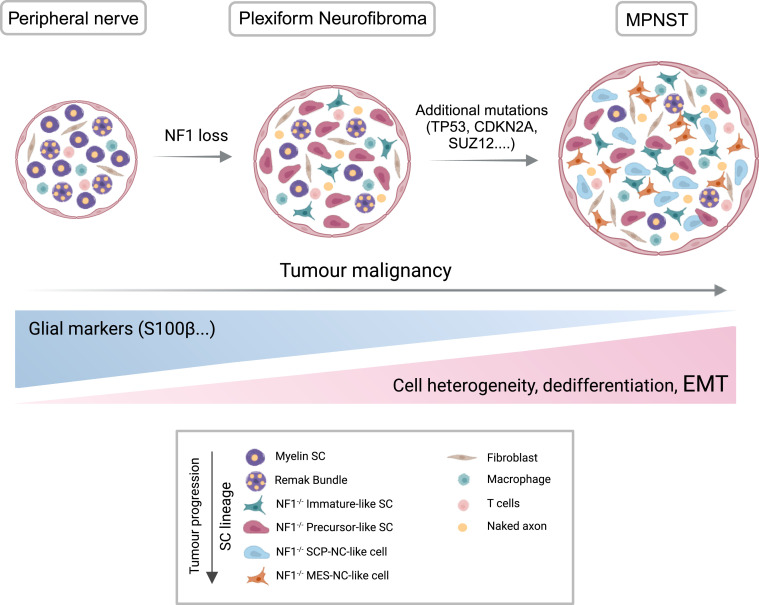
Role of EMT in SC reprogramming and tumour cell plasticity during NF development, and the progression from NF to MPNST. Oncogenic events like NF1 loss in the SC lineage can lead to the development of benign NF tumours. Additional mutations such as those occurring in TP53, CDKN2A or PCR2 components (SUZ12) in SCs promote benign NF transformation into malignant MPNST. The malignancy of these tumours is linked to cellular plasticity and heterogeneity that result from the induction of the EMT in SCs. In advanced stages, new dedifferentiated and malignant SCP/NC-like and MES-NC-like tumour cells emerge with a strong EMT and the loss of glial cell markers such as S100β (figure created with BioRender.com).

PNs may undergo further transformation into malignant peripheral nerve sheath tumours (MPNST). MPNST is a highly aggressive soft tissue sarcoma that represents the main cause of death in patients with NF1 [151]. MPNSTs are among the most difficult mesenchymal malignancies to treat [152]. The malignancy and the resistance to treatments of these tumours are often linked to cellular plasticity and heterogeneity that result from EMT induction (figure 3). EMT provides malignant traits to benign tumour cells, such as enhanced migration, invasion and chemoresistance [153]. Interestingly, loss-of-function mutations in TP53, CDKN2A or in the polycomb repressive complex 2 (PRC2), an epigenetic regulator associated with EMT in other contexts and tumour types [154–156], trigger PN progression into MPNST [157–160]. For instance, PRC2 through the interaction with EMT-TFs, such as Snail1 or Zeb2, represses epithelial gene expression, allowing the activation of mesenchymal traits that finally promote metastasis [155]. Particularly, in MPNST, the loss of the PRC2 subunit SUZ12 potentiates the effect of NF1 mutations by amplifying Ras-driven transcription, which, in turn, induces PN malignant transformation [161]. Moreover, loss of the PRC2 subunits Suz12 or EED leads to changes in cellular plasticity promoting a neural crest-like state that enhances metastatic potential [156]. In line with this, cellular heterogeneity increases over the course of tumourigenesis from PN to MPNST. In advanced stages of peripheral nerve tumours, new mesenchymal neural crest-like tumour cells (MES-NC-like cells) emerge. These cells mimic embryonic NCCs with a strong EMT signature including Zeb1 expression and the loss of normal SC lineage fidelity [162]. In anatomically distinct dermal neurofibromas, similar phenotypic changes and the emergence of these new MES-NC-like tumoural cells are also detected. Moreover, in MPNST and not in an early neoplastic state, malignant SCPs show a hybrid signature of NCC with a strong EMT (SCP-NC-like cells). In addition, the malignant NC-like cells that are also present in the tumour acquire traits similar to those of pre-migratory NCCs (figure 3).

Interestingly, scATAC-seq analysis demonstrates that the MES-NC-like cell population exhibits accessible chromatin regions enriched in genes associated with embryonic neural crest development (Six1, Zic1 and Zic2), EMT inducers (Zeb1, Snail2 and Tgfb2) and mesenchymal traits (Msx1, Meox1 and Eya1). In agreement with this, all the SC-derived from advanced-stage tumours increase the expression of cancer invasion genes and EMT-related genes such as Igfbp2, Ctgf and Egr1 [162]. In fact, these malignant SCs are transcriptionally similar to SCs that are located in the nerve bridge upon injury [19], which display a significant mesenchymal phenotype and EMT signature. Consequently, it is proposed that the oncogenic transformation of SCs, at least in part, mimics the transcriptional processes of SCs reprogramming during nerve repair. However, this transcriptional reprogramming is not sufficient for SCs to become oncogenic, as demonstrated by elegant lineage-tracing studies [3,163]. Indeed, additional events are required to trigger tumour formation, including mutation-driven inactivation of tumour suppressor genes as mentioned above [157–160], its silencing through promoter hypermethylation [164] or the downregulation of the lnk4a/Arf locus expression to prevent the senescence programme, leading to uncontrolled proliferation in reprogrammed SCs [165]. Moreover, as it occurs in PNs, PTPRS expression is also frequently reduced in MPNST, which significantly enhances migration and invasion of the tumour cells by promoting EMT processes [166]. All these studies indicate the pivotal role of EMT in SC reprogramming and tumour cell plasticity during NF development, the transition from NF to MPNST and its progression.

### 4.3. Schwann cells in the tumour microenvironment

The tumour microenvironment (TME) is a complex milieu comprising various cell types and signalling molecules. It plays a critical role in the development and progression of tumours and has emerged as a pivotal area of current cancer research [167,168]. Among the components of the TME, the PNS has been recently reported to be involved in the progression and dissemination of various types of cancers [169,170]. Although the lack of reliable models has resulted in a paucity of studies on neural niches in the TME, recent evidence shows that SCs are key elements in regulating tumour development and progression [171–173]. In response to nerve injury caused by cancer cells, SCs can acquire repair phenotypes and functions similar to those in the nerve regeneration process through adaptive reprogramming, which is closely linked to increased innervation in cancer [174]. SCs within the TME express a specialized regenerative ECM [175], ECM degrading enzymes and neurotrophic factors and play a role in immunoregulation [172], tumour related-pain [176] and nerve remodelling [177,178].

In certain tumours, reprogrammed SCs are able to contact tumour cells from primary tumours and enable their migration towards the nerve, promoting what is known as perineural invasion (PNI) [179–181], which is associated with tumour aggressiveness and increased metastasis [182]. As occurs after nerve injury, SCs within the TME induce the expression of molecules that can activate EMT signatures in cancer cells and therefore enhance their migratory and invasive properties. As such, recent research has demonstrated that EMT is an important programme involved in the PNI process in some neurotrophic cancers (table 1). In pancreatic ductal adenocarcinoma (PDAC) where most of the studies investigating SCs’ role in the TME have been performed, Tian *et al*. [183] showed that SC transcriptional reprogramming is associated with the upregulation of a series of cytokines that promote cancer cell migration and PNI. In particular, this study shows that SCs-derived CCL7 binds to its receptor CCR2 in cancer cells to upregulate the expression of the EMT-related factor TIMP1, while TIMP1 binds to CD63 to enhance the expression of CCL7 in SCs. As a proinflammatory cytokine, CCL7 promoted PDAC invasion and migration through the EMT induction by enhancing the expression of Timp1 and consequently Twist1, vimentin, N-cadherin and Snail1 [183]. Another mechanism that has been reported to promote pancreatic cancer cell invasion and metastasis is the activation of the nuclear factor kappa B (NFkB/p65) pathway in SCs by tumour-secreted IL1B. The activation of NFkB in SCs increases the secretion of IL6 that activates STAT3 signalling in cancer cells, leading to EMT induction with the upregulation of genes such as N-cadherin and Snail1 [184].

**Table 1.** Summary of different mechanisms by which SCs present in the TME induce EMT in cancer cells.

type of cancer	cytokines released by SCs in the TME	pathway activated in tumour cells	EMT changes in tumour cells	enhanced tumour capabilities	reference
PDAC	CCL7	CCR2 receptor	↑ Timp1 ↑ Twist1 ↑ Vimentin ↑ N-cadherin ↑ Snail	PNI migration	[183]
PDAC	IL6	STAT3 signalling	↑ N-cadherin ↑ Snail1	PNI metastases formation	[184]
salivary adenoid cystic carcinoma	BDNF	TrkB receptor	↓ E-cadherin ↑ N-cadherin ↑ Vimentin	PNI	[185]
colon cancer	NGF	TrkA/ERK/ELK1/ZEB1 signalling	↑ Vimentin ↑ N-cadherin ↑ Zeb1	PNI metastases formation	[186]
lung cancer	CXCL5	CXCR2/PI3K/AKT/GSK-3β signalling	↑ Snail1 ↑ Twist	migration invasion	[187]
cervical cancer	CCL2	CCR2 receptor	↑ Snail1 ↑ Twist	PNI migration	[188]
cholangiocarcinoma	TGFβ	TGFBR1 receptor	↓ E-cadherin ↑ N-cadherin	PNI proliferation	[189]
PDAC	TGFβ	TGFβ/SMAD signalling	↓ E-cadherin ↓ F-actin ↓ β-catenin	PNI migration	[190]

Regarding other tumour types, Shan *et al*. [185] established a co-culture model of salivary adenoid cystic carcinoma cells (SACC) and SCs to investigate molecular mechanisms involved in PNI. They reported that SCs in contact with tumour cells elevate BDNF levels, which binds to its receptor TrkB that is highly expressed in SACC cells. Consequently, EMT was induced in tumour cells and resulted in E-cadherin downregulation and N-cadherin and vimentin upregulation, which enhances the PNI process [185]. Similarly, SCs-derived NGF activates TrkA/ERK/ELK1/ZEB1 signalling in colon cancer cells inducing the expression of mesenchymal markers such as vimentin, N-cadherin and Zeb1 and promoting tumour invasiveness and metastasis formation [186]. This enhancement of the migration and invasion properties has also been reported by Zhou *et al*. [187] in lung cancer [187]. In this case, SC-derived CXCL5 by binding to its receptor CXCR2 in tumour cells activates PI3K/Akt/GSK-3β signalling, increasing the expression of the EMT-TFs Snail1 and Twist1 in these cells [187] and also in hepatocellular carcinoma cells [191]. In the context of cervical cancer, SCs secrete the cytokine CCL2 to promote PNI through the activation of EMT in tumour cells. In particular, CCL2 binds to its receptor CCR2 and induces the expression of EMT-TFs Snail1 and Twist1 in cancer cells, producing morphological changes to support tumour cell migration toward SCs [188]. Co-culture experiments also showed that SCs can induce EMT in cholangiocarcinoma (CCA) cell lines that appear to be TGFβ-dependent. Mechanistically, TGFβ released by SCs binds to TGFBR1 triggering a cadherin switch with the decrease of E-cadherin and the increase of N-cadherin in CCA cells [189]. This feature, commonly observed in TGFβ-induced EMT transition, confers aggressive properties to CCA cells that become more proliferative and invasive *in vitro* [192]. A similar effect is observed in pancreatic cancer cells, where TGFβ secreted by SCs was sufficient to induce the TGFβ/SMAD signalling pathway that reduces the expression of F-actin, β-catenin and E-cadherin in PDAC cells promoting tumour aggressiveness (migration, invasion and tumorigenicity) *in vitro* [190].

Besides studies regarding the ability of SCs to increase tumour cell invasion and PNI, it has been reported that SCs are able to promote PDAC progression *in vivo* by shaping the phenotype of tumour cells from the classical-like subtype, which exhibits higher expression of the epithelial markers, to a basal-like subtype [193]. This basal-like phenotype, which is widely recognized as a highly aggressive subtype in PDAC [194,195], has elevated expression of EMT-related genes and cell cycle pathways when tumour cells are treated with conditioned media from SCs [193].

All this suggests that reprogrammed SCs induce EMT in cancer cells to promote its progression; however, whether EMT activation in SCs from the TME regulates tumour biology remains to be investigated.

## 5. Conclusions and future perspective

SCs are derived from NCCs and exhibit significant plasticity, which is essential for development and injury responses. This adaptability is largely attributed to their capacity to reprogramme and transition through distinct phenotypes by activating signalling pathways like EMT. During development, TFs regulating EMT, such as Zeb2, are crucial for the lineage of SCs. The exact role of EMT in both myelinating and non-myelinating Remak SCs is poorly understood. Myelinating SCs are necessary for the development of the myelin sheath, while Remak cells support small unmyelinated fibres. Despite the different morphological and functional properties of these two types of SCs, the role of EMT-driven plasticity and the extent to which this process contributes to their adaptation during development is not clearly defined. However, the role of EMT extends beyond developmental stages. In adults, quiescent but plastic SCs activate EMT to reprogramme into a repair phenotype during tissue injury, enhancing cellular motility, morphological flexibility and cell cycle re-entry for effective tissue regeneration.

Nevertheless, the activation of EMT in SCs seems to have a dual role. While it is beneficial in promoting nerve regeneration, it can also contribute to pathologies. For example, during infections with intracellular pathogens like ML, SC plasticity can be exploited to aid bacterial dissemination. Similarly, in cancer, aberrant EMT activation in SC-derived tumours increases cellular plasticity and heterogeneity, promoting malignancy and tumour progression. In other tumours, such as prostate or pancreatic cancer, SCs in the TME acquire activated phenotypes that contribute to tumour migration and aggressiveness through the partial activation of the EMT in cancer cells.

**Figure 4.**
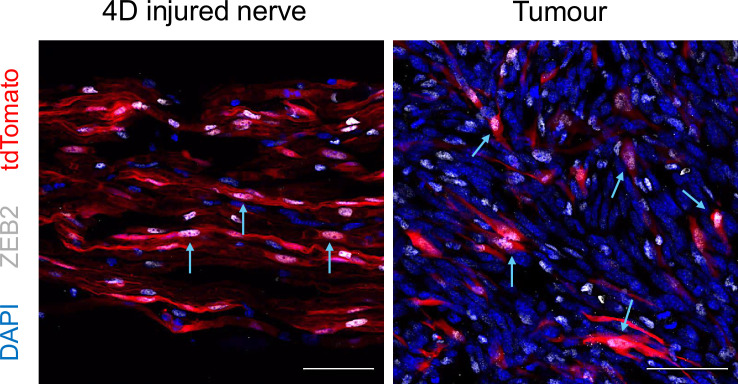
Reactivation of the EMT-TF ZEB2 in injury-activated SCs. Representative images of immunolabelling for ZEB2 in sciatic nerves 4 days after injury (left panel) and in syngeneic tumours growing upon injection of melanoma cells (right) in Plp1-CreER^T2^; tdTomato mice. Traced SCs are labelled with red and nuclear Zeb2 expression in white. Scale bar, 50 µm. Blue arrows indicate Zeb2^+^-SCs.

Although the role of EMT in SCs during physiological processes is increasingly understood, its involvement in pathological conditions like cancer remains less clear. For instance, it is well-known that the EMT-TF Zeb2 is crucial for SC differentiation and nerve remyelination; however, future studies are needed to determine how reactivation of Zeb2 and other EMT inducers regulate SC plasticity in MPNST or when SCs are present in the TME and how they might be targeted to prevent cancer progression (figure 4). This will enable the identification of novel key factors, including glial cell-derived neurotrophic factors, neural growth factors, chemokines and EMT-related factors, which can be specifically targeted to block tumour progression and metastasis formation.

Given the tightly regulated nature of SCs reprogramming by mechanisms such as EMT, there is potential for developing pharmacological or genetic interventions to manipulate these cells. From a clinical perspective, this would be a significant advancement as there is a clear need to enhance the repair-supportive functions of reprogrammed SCs. However, the challenge lies in selectively modulating EMT processes to maximize regenerative benefits while minimizing adverse effects in pathological conditions. Therefore, further research is essential to fully elucidate the specific roles and regulation of EMT in SC plasticity across developmental, regenerative and disease contexts.

## Data Availability

This article has no additional data.
